# Individualized Stress Mobile Sensing Using Self-Supervised Pre-Training

**DOI:** 10.3390/app132112035

**Published:** 2023-11-04

**Authors:** Tanvir Islam, Peter Washington

**Affiliations:** Information and Computer Sciences, University of Hawaii at Manoa, Honolulu, HI 96822, USA;

**Keywords:** mobile sensing, affective computing, personalized machine learning, self-supervised learning, biosignals, stress prediction

## Abstract

Stress is widely recognized as a major contributor to a variety of health issues. Stress prediction using biosignal data recorded by wearables is a key area of study in mobile sensing research because real-time stress prediction can enable digital interventions to immediately react at the onset of stress, helping to avoid many psychological and physiological symptoms such as heart rhythm irregularities. Electrodermal activity (EDA) is often used to measure stress. However, major challenges with the prediction of stress using machine learning include the subjectivity and sparseness of the labels, a large feature space, relatively few labels, and a complex nonlinear and subjective relationship between the features and outcomes. To tackle these issues, we examined the use of model personalization: training a separate stress prediction model for each user. To allow the neural network to learn the temporal dynamics of each individual’s baseline biosignal patterns, thus enabling personalization with very few labels, we pre-trained a one-dimensional convolutional neural network (1D CNN) using self-supervised learning (SSL). We evaluated our method using the Wearable Stress and Affect Detection(WESAD) dataset. We fine-tuned the pre-trained networks to the stress-prediction task and compared against equivalent models without any self-supervised pre-training. We discovered that embeddings learned using our pre-training method outperformed the supervised baselines with significantly fewer labeled data points: the models trained with SSL required less than 30% of the labels to reach equivalent performance without personalized SSL. This personalized learning method can enable precision health systems that are tailored to each subject and require few annotations by the end user, thus allowing for the mobile sensing of increasingly complex, heterogeneous, and subjective outcomes such as stress.

## 1. Introduction

Chronic stress can drastically affect an individual’s health across several dimensions. Continuous stress increases the risk of cardiovascular disease, hypertension, reduced immunity, and cancer [1]. Despite these detrimental physical effects, stress is often left unmanaged. A growing body of work in the field of human–computer interaction (HCI) is focusing on mobile sensing of stress using signals from wearable consumer devices [2]. Real-time detection of stress via wearable devices can enable stress interventions, which can provide therapeutic support via immediate prompting when stress is detected. Until recently, these mobile sensing methods of subjective outcomes have required large amounts of training data from each user, making the deployment of such models infeasible in practice. We present a novel methodology for personalizing mobile sensing models using a minimal number of human labels.

Consumer wearable devices offer a range of biosignal measurements, which may include, but are not limited to, electrodermal activity (EDA), electrocardiograms (ECGs), electromyography (EMG), respiration rate, and galvanic skin resistance (GSR). EDA is a physiological measurement of the electric current that flows through the skin. EDA is particularly sensitive to changes in skin ionic conductivity caused by even minor perspiration that is not visible on the surface of the skin [3]. In comparison to other physiological measurements like heart rate variations and blood pressure, EDA is a robust way to gauge physiological arousal and, by extension, the response to stress. In the field of affective computing, EDA is widely recognized as a key marker of physiological arousal and stress responses [4]. Therefore, an EDA-based stress quantification approach was the primary focus of this investigation.

Deep neural networks (DNNs) have been a driving force behind major machine learning progress in recent years [5]. DNNs are multi-layer computational models that may learn progressively complex and high-level representations of the input data to accomplish a prediction goal. DNNs are an especially promising solution for analyzing biosignals due to their ability to spot patterns and learn meaningful features from unprocessed data inputs without requiring extensive data preprocessing or manual feature engineering [6].

Creating a stress prediction model using biosignal data that is generalizable is impractical due to the varying physiological responses to stress among individuals. As a result, stress-monitoring systems must possess the flexibility to accommodate these differences [7]. Rather than building a one-size-fits-all stress-prediction model, we propose a novel approach aimed at developing individual-specific models, with the goal of achieving greater personalization. Such models can enable precision health mobile sensing systems to reach clinically useful performance.

A major hurdle in developing personalized mobile sensing models through biosignal data is the procurement of adequate supervisory data (i.e., ground truth labels) to train DNNs, which require large training sets for the training process to converge. Self-reported stress labels are often generated by laborious and faulty manual human labeling owing to human subjectivity and weariness [8].

Current research on EDA-based stress assessment tends to focus on supervised deep learning. Achieving clinically useful model performance is hindered, however, by the fact that traditional supervised learning does not make use of all of the unlabeled data that are available. Individuals who use wearable devices have copious amounts of unlabeled longitudinal body signal data, but only a few labels to indicate important health events. These labels are used for training machine learning models to recognize the events of interest.

To bridge this gap, we propose the use of self-supervised Learning (SSL) to learn neural network weights, which are optimized to make predictions about the baseline temporal dynamics of a user’s biosignals. Such weights can then be efficiently fine-tuned to personalized prediction tasks using that user’s biosignal data. We hypothesized that by utilizing self-supervised learning (SSL) on biosignal data from wearables, we can develop personalized stress prediction models that are more effective and efficient than traditional supervised learning models, even with limited labeled data. We implemented an instantiation of this SSL paradigm and, then, fine-tuned the resulting models to the stress-prediction task using EDA signals. In particular, we made the following contributions:

- We introduce a novel “personalized SSL” paradigm to address the common real-world situation of large datasets with few training labels. This solution enables mobile sensing systems to predict traditionally subjective classes such as stress. This interaction paradigm is similar to how Apple’s Face ID works: once the face recognition system is provided with only a few examples provided by the end users, it can be used repeatedly to unlock the device. In this case, the labels are stress predictions, rather than identity predictions, and the model input is wearable biosignals, rather than a face image.
- We evaluated this novel mobile sensing methodology on a real-world biosignal dataset with stress labels: the Wearable Stress and Affect Detection (WESAD) dataset.

Once the model is calibrated, it can be used repeatedly to predict how stressed that person will be in different scenarios. The stress-prediction task requires less computing power because the model uses the model weights learned during the training process to efficiently fine-tune towards stress prediction. This reduced need for computing resources means that the model can be processed faster, making it easier to use in real-world settings.

The remainder of the paper is structured as follows: Section 2 explores contemporary SSL techniques with bio-signals. Section 3 outlines our proposed method and its implementation. Section 4 presents the model results and performance. Section 5 discusses the strengths, limitations, and future directions. Section 6 provides concluding remarks.

## 2. Related Work

### 2.1. Stress Assessment Using Biosignals

The hyperactivity of the nervous system is associated with chronic stress and is responsible for its wide range of adverse effects on health and behavior. Stress and brain activity are closely correlated [9]. Hannun et al. showed that single-lead ECGs can be used to identify rhythms using an end-to-end deep learning method. They demonstrated that the 1D CNN model architecture can be successfully applied towards making predictions from biosignal data without manual feature engineering and feature selection [6].

Variations in the skin’s electrical characteristics are reflected by EDA, making it a helpful stress indicator for the nervous system. Sweating is a natural response to stressful situations and may further enhance the skin’s conductivity [10]. Spathis et al. described a stress-prediction framework that enables consumers to receive feedback on their stress levels through speech-enabled consumer devices like smartphones and smart speakers [11]. EDA sensors are located on the hands, whereas ECG sensors are located on the chest. Data from the EDA sensors are wirelessly transferred to the ECG sensor through WiFi. This method attempts to improve stress prediction by capturing two EDA channels to eliminate motion artifacts. The designed sensor yields excellent linearity and jitter performance and exhibits minimal delays during wireless transmission. Zhu et al. studied the possibility of detecting stress using EDA data acquired from wearable sensors [12]. The study made use of two publicly accessible datasets: VerBIO [13] and WESAD [14], both of which comprise EDA signals from research-grade wearable sensors. The experimental results demonstrated that Random Forests differentiate stress from non-stress conditions with an accuracy of 85.7%, highlighting the potential for wearable devices with EDA sensors to anticipate stress. The study also evaluated the dependability of training with extracted statistical and EDA-related features from EDA signals, discovering no consistent trend between employing complete features and chosen features. The findings indicated the effectiveness of placing EDA sensors into wearable devices for stress prediction, instilling confidence that incorporating EDA sensors into smartwatches is a potential option for future product development.

In addition to research-grade equipment, EDA is also frequently measured using consumer wearable devices. Using low-resolution electrodermal activity (EDA) data from consumer-grade wearable devices, Ninh et al. explored the stress prediction capabilities of user-dependent and user-independent models [15]. This work revealed that stress-prediction models employing low-resolution EDA data from wrist-worn and finger-mounted sensors have high balanced accuracy scores (66.10% to 100%). This finding indicated that low-resolution EDA signals from consumer-grade devices may be used to efficiently create user-dependent stress-prediction models, enabling the collection of in-the-wild data for mental health tracking and analysis.

EDA is often used in conjunction with other biosignals. Eren et al. suggested a way to detect stress by using sensors from the Empatica E4 device used in the WESAD [14] dataset to measure blood volume pulse (BVP) and electrodermal activity (EDA) [16]. The suggested neural network model was 96.26% accurate. The study also called for more research to test the method’s usefulness and general flexibility. Pakarinen et al. [17] explored the capacity of electrodermal activity (EDA) to precisely categorize mental states, including relaxation, arousal, and stress, in addition to self-perceived stress and arousal, utilizing a three-phase modified MIST test. The research demonstrated high levels of accuracy in identifying these mental states, indicating that EDA has the potential for long-term evaluation of stress and arousal in the workplace. They explored the utilization of machine learning models for stress detection and classification, employing features derived from physiological signals, including EDA and BVP. By achieving an F1-score of 0.71, the study highlighted the potential to create a seamless, real-world stress-monitoring system and promoted continued investigation through the integration of machine learning and signal-processing methodologies in a professional context. Kalimeri et al. proposed an advanced multimodal framework to assess the emotional and cognitive experiences of visually impaired individuals while navigating unknown indoor environments, leveraging mobile monitoring and the combination of EEG and EDA signals [18]. The goal was to identify the environmental factors that increase stress and cognitive load, thereby guiding the creation of emotionally intelligent mobility systems that can adapt to challenging environments using real-time biosensor information.

### 2.2. SSL on Biosignals

The field of SSL on biosignals is an emerging field of study. Two popular SSL approaches, SimCLR [19] and BOYL [20], are contrastive learning approaches, which rely on data augmentation. This methodology contains limitations such as invariance, which can obscure valuable detailed information necessary for specific downstream tasks. Additionally, predicting which augmentations will yield optimal benefits can prove to be challenging [21]. As a result, in our study, we excluded the use of data augmentation and chose instead to focus on pre-training and downstream tasks with raw data. Pöppelbaum et al. introduced a novel self-supervised learning approach for time series analysis using the SimCLR contrastive learning model, which was enhanced with new data augmentation techniques. The authors demonstrated improved fault classification accuracy [22]. Robert et al. offered a contrastive-learning-based stress-prediction system that uses modified EDA signals to classify stress and non-stress circumstances [23]. Nevertheless, the current project differs from this approach because it did not require any signal transformation. Instead of only stress vs. non-stress categorization, our presented study allowed for the learning of a wide range of stress levels. By using the temporal and spatial information included within ECG impulses, CLOCS uses contrastive learning to acquire unique representations of each patient [24]. This novel concept considers a positive pair as a representation of two changed occurrences that both correspond to an identical patient. As a result, the model automatically adjusts the learned representations to the specifics of every patient’s case. Sarkar et al. offered a self-supervised method for learning ECG representations for the purpose of emotion classification [25]. Eldede et al. introduced a novel time series representation learning framework, TS-TCC, leveraging contrastive self-supervised learning for unlabeled data, focusing on temporal and contextual contrasting [26]. Additionally, they extended their model for semi-supervised settings and demonstrated its effectiveness through extensive real-world dataset experiments. Yang et al. proposed TimeCLR, a self-supervised representation learning framework tailored to univariate time series, which integrates the benefits from dynamic time warping (DTW) and InceptionTime, offering improved performance in scenarios with limited labeled data across various time series domains [27]. Sunmin et al. propose SIM-CNN, a self-supervised learning framework that improves stress recognition by training a personalized multimodal CNN on a large dataset of nurses’ biosignals, both labeled and unlabeled, optimizing for individual physiological differences [28]. Tanvir et al. show by leveraging self-supervised learning on wearable biosignal data, personalized multimodal stress prediction system outperforms traditional supervised models with minimal manual annotations, addressing the challenges of label scarcity and biosignal heterogeneity [29].

Table 1 provides a summary of the literature review.

## 3. Methodology

**Figure 1.**
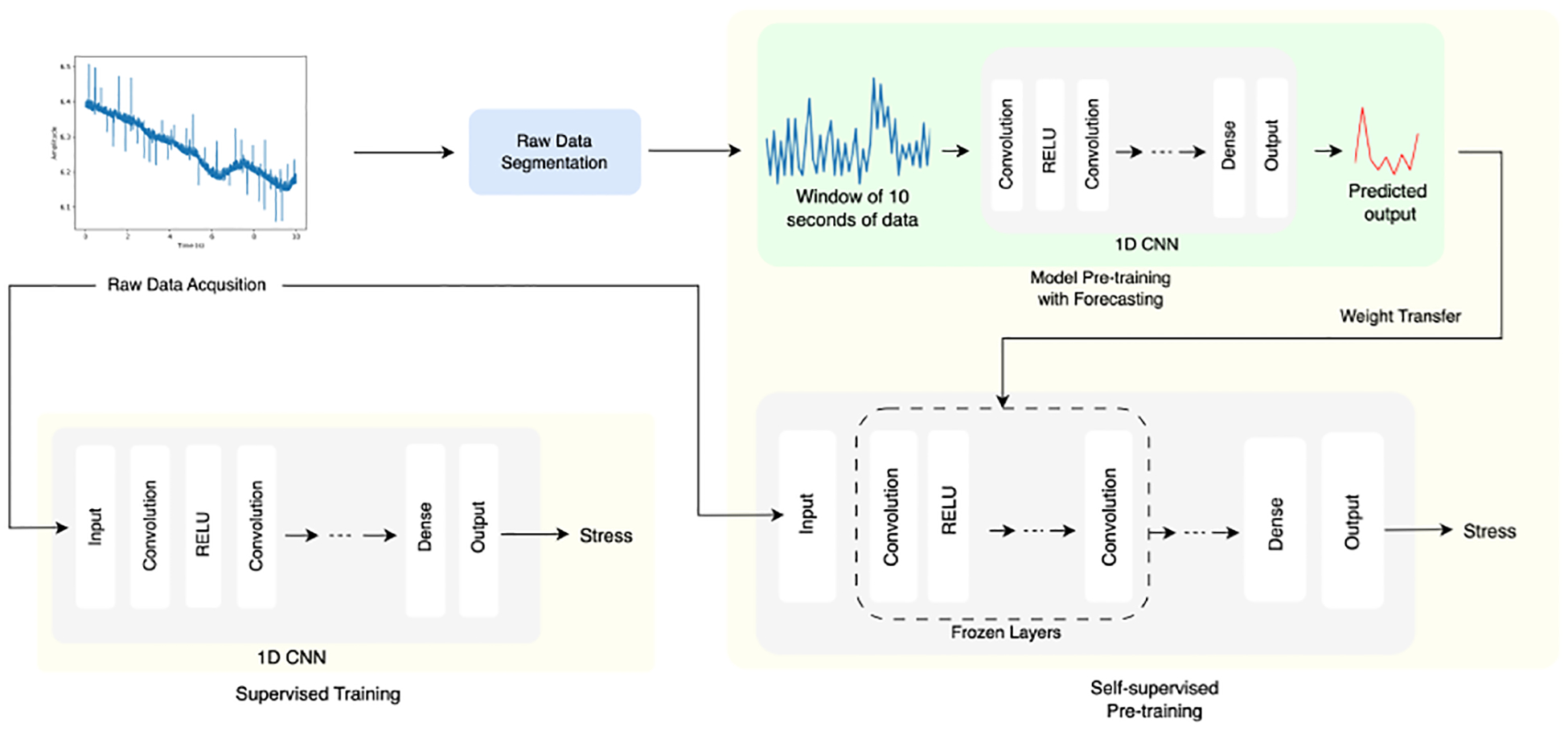
Overall process of our personalized stress-prediction framework. The raw signal is segmented into distinct, but overlapping data points. A forecasting model is used for self-supervised pre-training. These weights are fine-tuned to the stress-prediction task (**right model**). We compared this model against a supervised learning model without self-supervised pre-training (**left**).

We propose a two-step process for training mobile sensing models, which can predict subjective labels such as stress using only a few training examples per user: (1) personalized SSL for representation learning of temporal EDA dynamics per user and (2) fine-tuning the pre-trained model to the stress-prediction task. During SSL, the pretext task helps the model learn interim data representations without ground truth labels. The pretext task is designed to predict the missing segments of the input data given the remaining segments. After this representation is learned, the downstream task involves fine-tuning the weights from the pretext task towards training the neural network model to predict stress. The overall architecture is shown in Figure 1.

As depicted in Figure 1, the personalized SSL model was designed to accept the raw EDA signal for each subject. The raw EDA signal is divided into chunks of 7000-dimensional vectors, as described in Section 3.3, generating a large dataset of 7910 data points to pre-train the model. The pre-training phase involved the use of a 1D CNN model to predict 40 data points, as detailed in Section 3.3. The weights learned in the pre-training phase are transferred to another 1D CNN model, which is fine-tuned for the stress-prediction task.

In addition, a second 1D CNN model with the same architecture as the fine-tuned model was employed for stress prediction, but without any pre-training. This is our control model, which we used as a baseline to quantify the benefits of our SSL over personalization without SSL.

We compared these two models using varying numbers of labeled data points, as described in Sections 4.2 and 4.3.

We performed a subject-dependent study by directly comparing the following two models trained and evaluated separately per subject:

- **Model A**: stress prediction via supervised learning;
- **Model B**: stress prediction with a model pre-trained with SSL and fine-tuned using the same supervised learning strategy as Model A.

### 3.1. Dataset

We used the WESAD [14] dataset to evaluate our methodology. The WESAD [14] dataset is a freely accessible multimodal physiological dataset containing ECG, EDA, EMG, respiration (RESP), core body temperature (TEMP), and three-axis acceleration (ACC). Biosignals were collected from 15 healthy adults (mean age 27.5; standard deviation (SD) = 2.4)) when they were subjected to one of three conditions: neutral, stress, or amusement, in a controlled laboratory setting. The researchers used the Trier Social Stress Test (TSST) to induce anxiety. Two devices: Empatica E4 and RespiBAN, were used to collect signals for this dataset. Using the RespiBAN device, the data were acquired from each participant for around 33 min at a sampling rate of 700 Hz. Multiple sensors, including ECG, EDA, EMG, RESP, TEMP, and ACC, are included in the RespiBAN device. The Empatica E4 records sensor data on BVP at 64 Hz, EDA at 4 Hz, TEMP at 4 Hz, and ACC at 32 Hz. Participants were asked six questions from the State-Trait Anxiety Inventory (STAI) to derive insight into their degree of anxiety. Each question was answered using a four-point Likert scale. The STAI questions are as follows:

- Question 1: I feel at ease.
- Question 2: I feel nervous.
- Question 3: I am jittery.
- Question 4: I am relaxed.
- Question 5: I am worried.
- Question 6: I feel pleasant.

In this study, we used stress data for the baseline condition only. Our models were trained using EDA signal data from the RespiBAN device with a sampling rate of 700 HZ.

### 3.2. Label Representation

We converted the categorical ordinal outcome variables (i.e., the Likert scale responses to the questionnaires) into a representation that maintained the categorical nature of the responses, as well as the ordinal nature. In the WESAD dataset, participants were given six questions from the STAI (see Section 3.1 for details). Participants were asked to rate this question on a four-point Likert scale. Therefore, each question was labeled between 1 and 4. To model the ordinal regression task, we converted the original labels of (1, 2, 3, 4) into the probabilities of (0.25, 0.5, 0.75, 1.0). Because the four-point Likert chart used in the original evaluation was equally spaced, these labels were quantified and distributed uniformly. These labels are subjective since the models use only within-subject data and participants are internally consistent in their labeling (i.e., we assumed that a rating of 3 consistently indicates higher severity than a rating of 2 on the same question within each participant’s set of ratings).

### 3.3. Self-Supervised Pre-Training

The use of self-supervised pre-training, an unsupervised approach in which data are self-labeled and, subsequently, fine-tuned using supervised learning approaches to extract important feature representations, has proven to be more effective than supervised training alone in an increasing number of applications [19]. The self-supervised pre-training approach focuses on learning a high-level representation of data without using ground truth labels, which is also known as the pretext task. A pre-trained model enables efficient transfer to a target task since the representation it has learned is already suited (or readily transferable) for a given job. SSL in this manner is the same technology that powers large-scale language models like ChatGPT and natural language training tasks powered by BERT [30].

We pre-trained our model through forecasting, a similar technique used to pre-train OpenAI’s GPT language model [31]. A separate model was pre-trained for each human subject using only the data from that subject. To pre-train our model, we employed a 1D CNN, a standard neural network architecture for signal prediction [32]. Prior research has demonstrated the robustness of the 1D CNN in 1D signal processing tasks, particularly in the domain of biosignals or 1D signals [6]. One of the key advantages of 1D CNNs is their ability to automatically learn relevant features from the input signal, eliminating the need for manual feature engineering. This can save time and effort while also improving the model’s performance, as it can adaptively learn the most-discriminative features for a given task.

**Figure 2.**
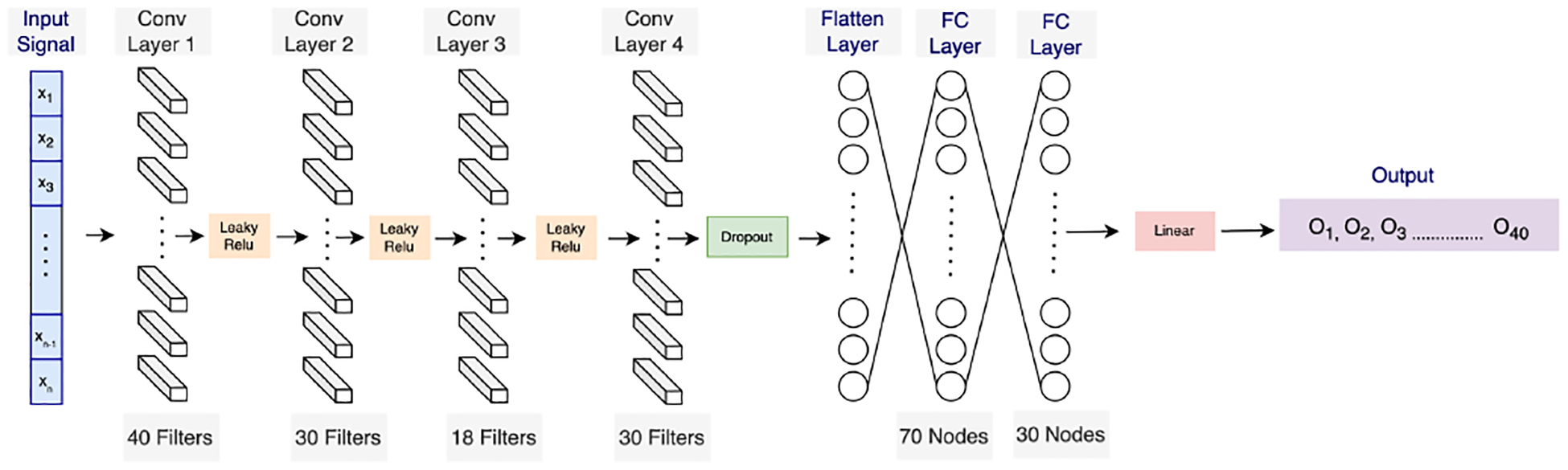
The 1D CNN architecture for the pretext task.

Moreover, 1D CNNs can capture information at different time scales due to the use of multiple neural layers and multiple independent learned kernels. This makes this family of architectures particularly helpful when dealing with signals with complicated hierarchical patterns or events that occur at different time scales. Additionally, 1D CNNs can handle input data with noise better than other machine learning algorithms, as the convolutional layers can learn to focus on the most-important features and ignore noise or irrelevant signals. Figure 2 illustrates the architecture that was used in this study for pre-training the model as a knowledge representation of the raw EDA signal. To maintain consistency, we used the same model architecture for both self-supervised pre-training and supervised training. We used the root-mean-squared error (RMSE) as our evaluation metric.

The pretext 1D CNN model was implemented with an input shape of (7000, 1), consisting of four 1D convolution layers, each utilizing a leaky ReLU activation function. The first convolution layer has 40 filters, followed by a layer with 30 filters, and then, a layer with 18 filters. The fourth convolution layer has 30 filters and uses a leaky ReLU activation function. Moreover, the model includes two dense layers, one with 70 nodes and the other with 30 nodes. The final layer is an output layer with a linear activation function. The convolution layers are responsible for extracting important features from the EDA signal, while the dense layers utilize these features to make a stress prediction.

**Figure 3.**
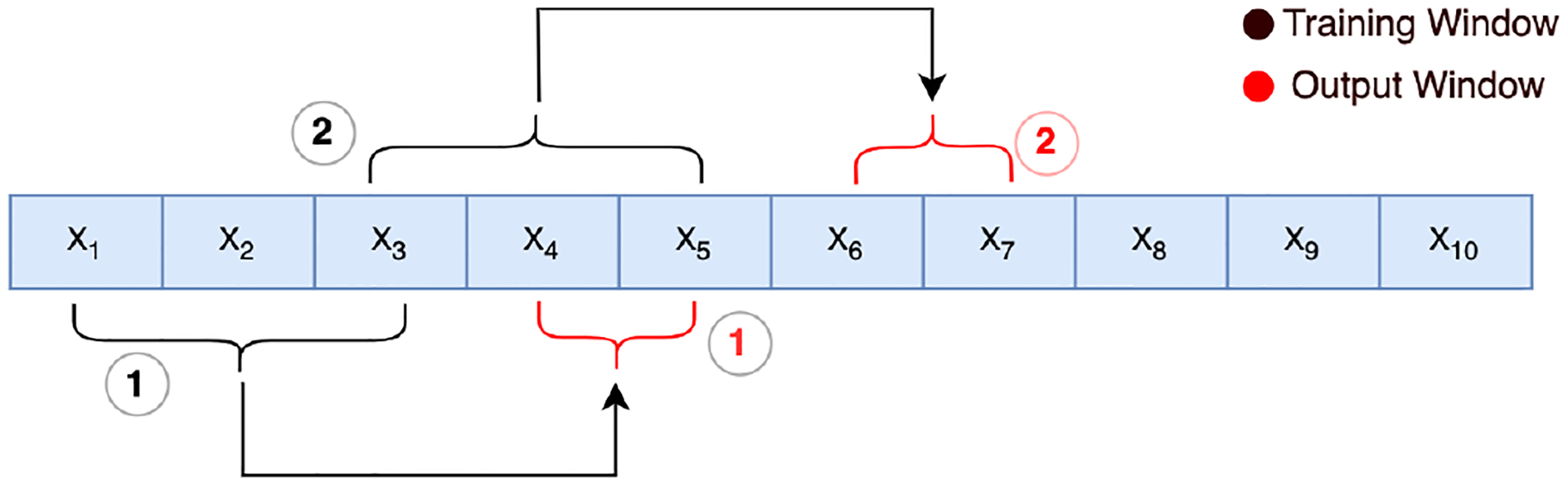
We used signal forecasting as our self-supervised pretext task. A sliding window was used to predict the following chunk of the EDA signal.

The model was pre-trained on the entirety of the EDA signal for each participant. We divided the whole signal into fixed windows (each containing a 7000-dimensional vector) (Figure 3). Figure 3 illustrates an example of the data points in the training set with overlapping information. We represent the signal as *X*_1_ ······ *X*_*n*_. Our method segments this signal into multiple fixed windows, each of length 7000 points. So, the first window captures the sequence *X*_1_, *X*_2_ ······ *X*_7000_. The purpose of this window is to predict the next 40 points, i.e., *X*_7001_, *X*_7002_ ······ *X*_7040_.

However, instead of progressing to a completely new window after this, we introduced overlap. The next window starts only 100 points after the beginning of the previous window, i.e., *X*_100_, *X*_101_ ······ *X*_7100_. Similarly, this window aims to predict the sequence *X*7101, *X*7102 ······ *X*7140.

By consistently overlapping windows by 100 points, we ensured that the model was exposed to various transitional patterns in the data. This overlapping approach continued until the end of the signal, resulting in a total of 7910 data samples for each participant. The motivation behind this strategy was twofold: it maximized the utility of available data and equipped the model to make stress predictions based on 10 s segments, offering a granular understanding of the participant’s stress evolution.

### 3.4. Downstream Stress-Prediction Task

Self-supervised pre-training offers the benefit of optimizing data efficiency during the fine-tuning process for subsequent downstream activities. The difficulty in sourcing high-quality labels makes this a critical scenario for use in mobile sensing research. We conducted a thorough quantitative analysis of this added benefit by contrasting the efficiency of freshly trained, solely supervised models against that of those pre-trained using self-supervised representations.

**Figure 4.**
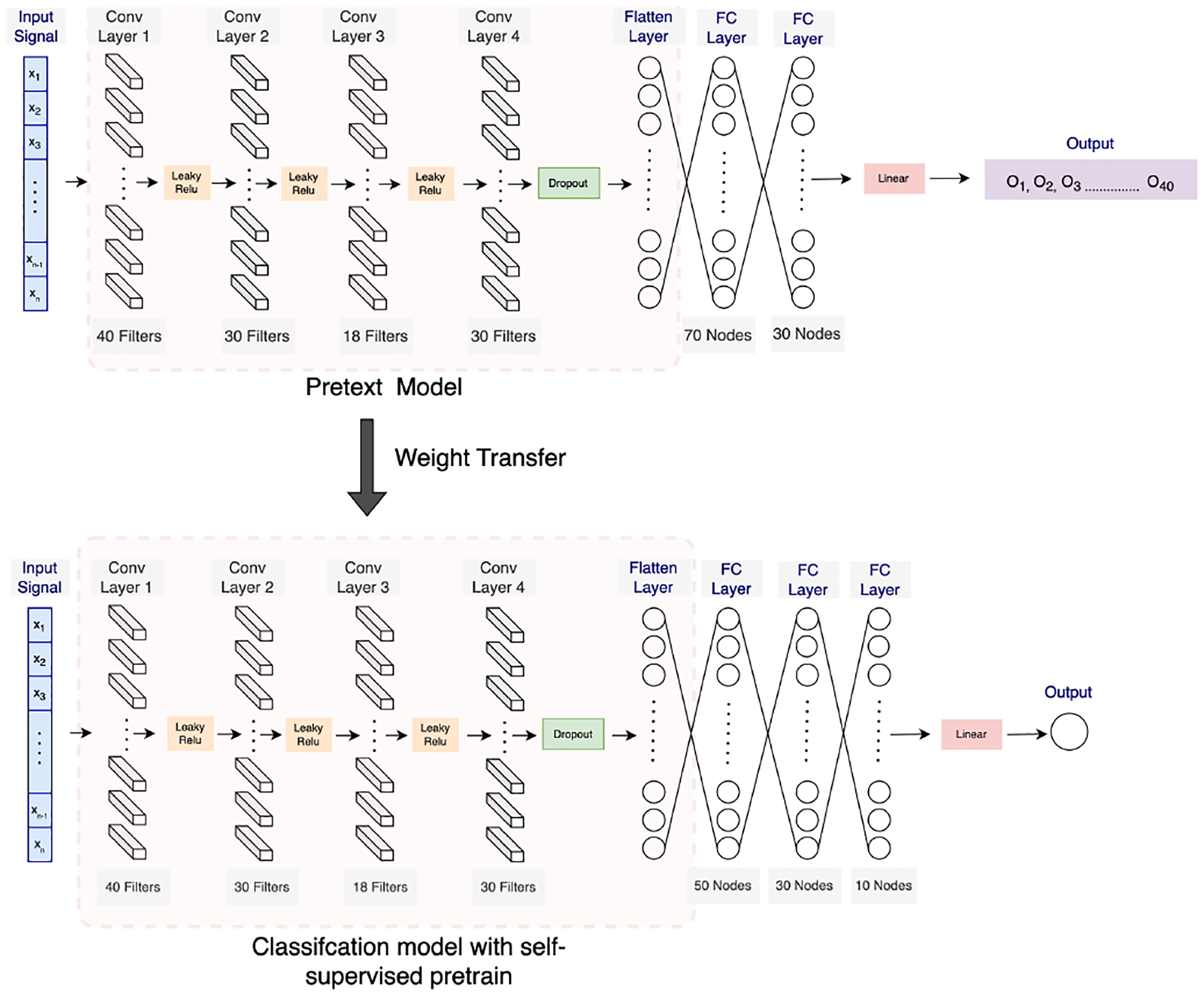
The 1D CNN architecture for fine-tuning the pre-trained network towards the downstream stress-prediction task.

In this stage, we used the same 1D CNN architecture that we used for model pre-training. However, in this architecture, the feature extractor layers were frozen, and the rest of the layers were fine-tuned to the stress-prediction task. For this downstream task, we modified the model with 3 new dense layers for the final prediction. The architecture for stress quantifying is shown in Figure 4. As illustrated in Figure 4, the fine-tuned model shares the same set of convolution layers and activation functions as the pretext 1D CNN model. However, it has three dense layers, the first one with 50 nodes, followed by a dense layer with 30 nodes, and the final dense layer with 10 nodes. The output layer has a linear activation function, and the predicted output is stress. When fine-tuning a pre-trained model, including extra fully connected (FC) layers has several benefits. Firstly, a pre-trained model often originates from a general or differing task. By integrating a new FC layer, the model acquires the capability to focus on patterns explicitly tailored for the new task, here stress prediction, facilitating superior feature extraction and representation for that particular task. Adding new FC layer ensures that the specific knowledge from the original pre-trained task does not dominate, offering the model a new layer to optimize and adapt to the unique features of the new task. This assures a more-harmonious transition and improved performance in the new task domain.

In the case of purely supervised training, the same architecture as the fine-tuned model was utilized to facilitate a direct comparison between the methods.

### 3.5. Experimental Procedures

For our experiment, we selected a fixed number of random data points having a window size of 7000, which corresponds to 10 s of data to train our model in both SSL and solely supervised manners with exactly the same set of data points. In both cases, we utilized the same model architecture and the same sampled data points. To train using SSL, we transferred the weights from the pre-trained model to the downstream task (Section 3.4). The average RMSE of the trained model using 10 data points is shown in Section 4.2. In this experiment, all models were subjected to an identical test set.

## 4. Results

We used the RMSE to evaluate our models, as regression models commonly utilize the RMSE as a statistic for assessing model performance. For a given test set, a model’s RMSE measures the average distance between the expected values and the actual values. It is measured as the square root of the average of the squared differences between the predicted and actual values. The RMSE is helpful because it penalizes large mistakes over smaller ones and quantifies far apart the mistakes in the original units of the dataset. A smaller RMSE indicates that the model performed with less error.

### 4.1. Pre-Trained Models

**Figure 5.**
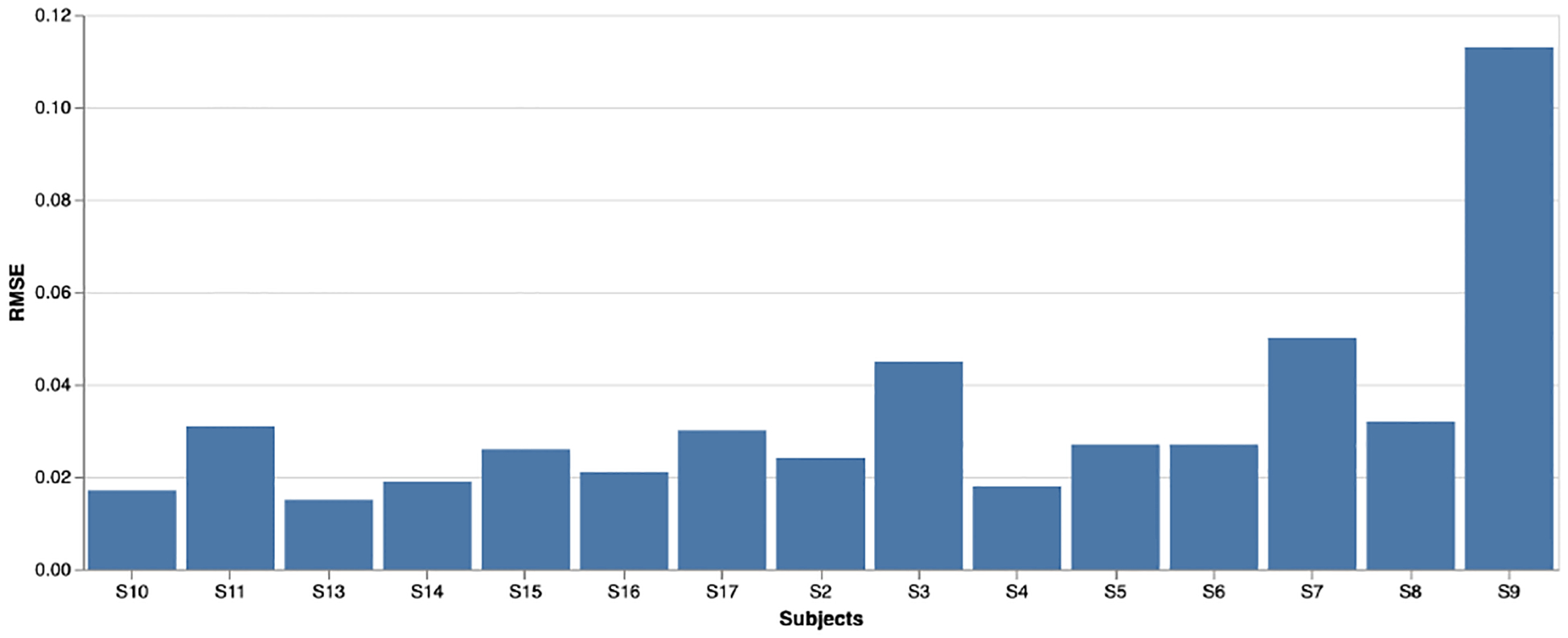
RMSE for the signal forecasting pre-training task for all subjects. The low RMSE indicates that the model learned the baseline dynamics of each user’s EDA biosignal.

For pre-training purposes, all the models were trained using data coming from a single subject. Figure 5 demonstrates the RMSE of the pre-trained models of all 15 subjects. The RMSE score is a measure of how well a model performed in predicting values compared to the actual values. For example, Subject 2’s pre-trained model achieved an RMSE score of 0.024, meaning that the model’s predictions were, on average, 0.024 units away from the actual values. In other words, the model’s predictions had an average error of 0.024 units. The lower the RMSE score, the better the model’s performance is. A smaller RMSE value indicates that the model is making more-accurate predictions, while a higher RMSE value indicates that the model is making larger errors. The low RMSE values we achieved across subjects suggested that our pre-trained models were well-trained and learned the underlying dynamics of each subject’s EDA signals.

### 4.2. Evaluation Using Pre-Trained Models

**Figure 6.**
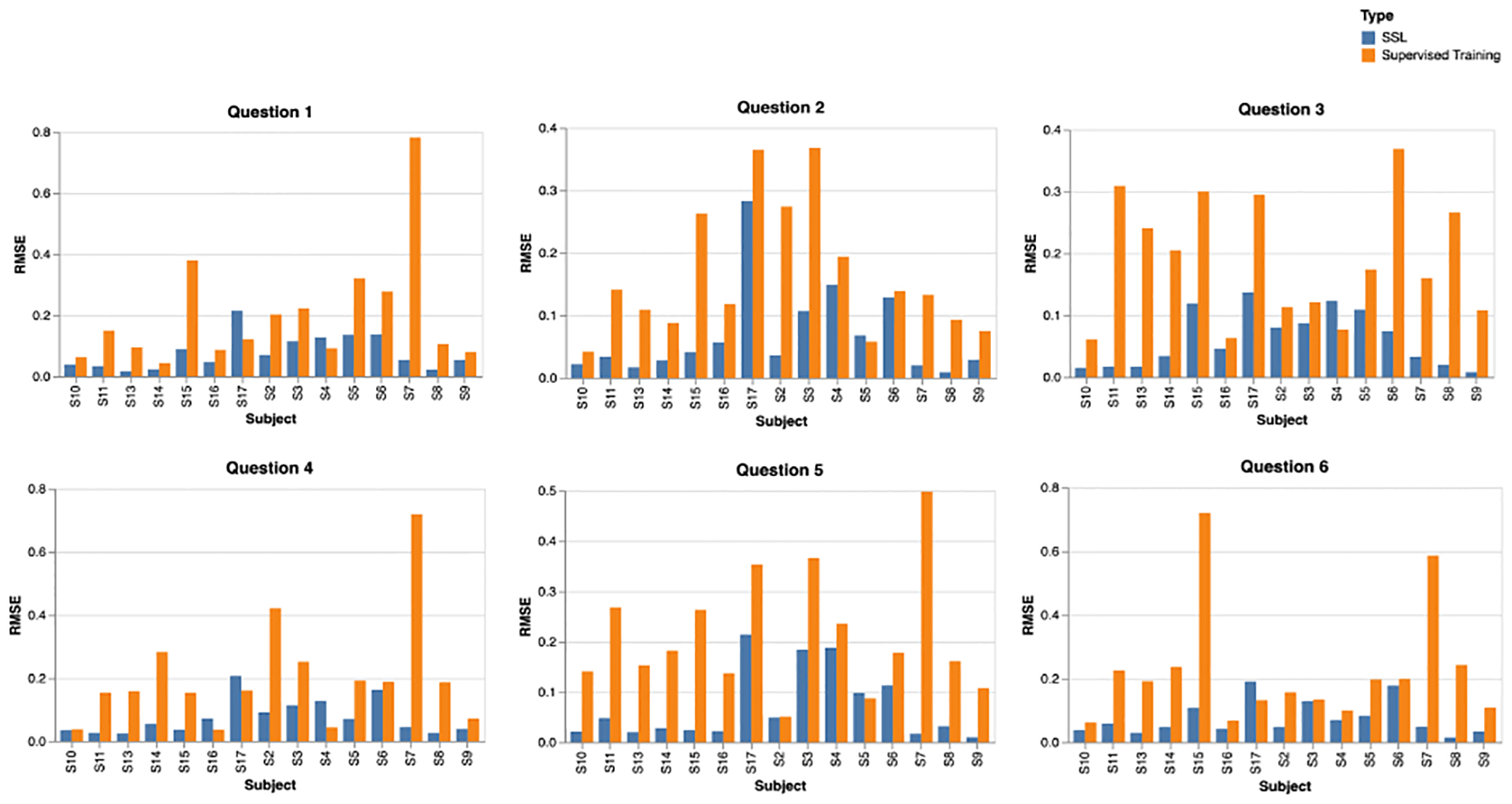
Comparison of the RMSE between a model fine-tuned after SSL vs. purely supervised training for all subjects and for all questions.

Figure 6 displays the RMSE score of all participants for each question in the WESAD dataset while training on a randomly sampled subset of 10 data points in both SSL and supervised modes. We conducted bootstrapped sampling using five independent samples, and the same samples were used to compare the SSL pre-trained and purely supervised models. From Figure 6, we observe that, in most of the cases, the RMSE score was lower for the SSL-based models than purely supervised trained models. We observed that the SSL models outperformed the purely supervised models in approximately 90% of cases.

Across the six questions, the performance metrics are depicted using RMSE values, comparing self-supervised learning (SSL) against purely supervised training. For Question 1, SSL excelled particularly with Subject S8, demonstrating a significantly lower RMSE. For Question 2, SSL showed more-favorable results for a majority of the subjects, especially for S2 and S3. For Question 3, the most-commendable SSL performance was observed for S7. Moving to Question 4, S8’s RMSE for SSL was notably efficient. For Question 5, both S3 and S8 stood out with SSL achieving lower RMSEs, while for Question 6, S7 was once again a highlight for SSL. Consistently, SSL demonstrated superior performance, with lower RMSE values being indicative of its effectiveness across subjects.

### 4.3. Results for a Demonstrative User

**Figure 7.**
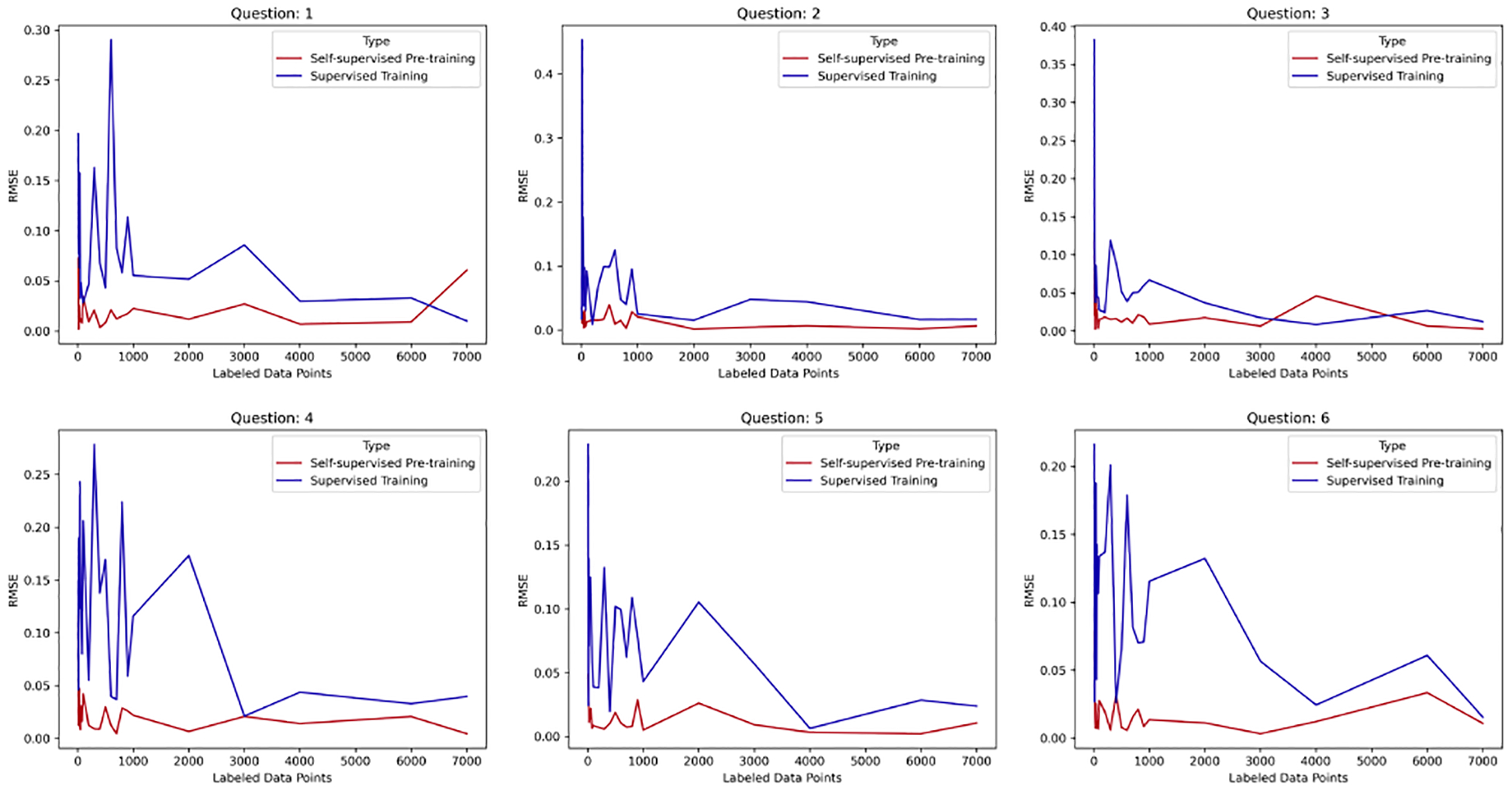
Comparison of the performance between SSL pre-training vs. purely supervised training for Subject 2 for all questions over different sets of labeled data points.

We compared purely supervised training and SSL, as shown in Figure 7. Our research indicated that the models employing SSL techniques required significantly less data to achieve comparable performance to those models that did not use SSL methods. We observed that the models that employed SSL techniques required less than 30% of the data needed by the models that did not use SSL techniques.

We saw that the models that only used fully supervised training methods needed more data to obtain the same RMSE as similar SSL models that were trained with the same number of labeled data points. This illustrates how SSL methods can speed up data-heavy applications by making them more efficient.

In addition to the benefits of preserving data, our findings demonstrated that SSL methods were stable across training runs. The RMSE of the purely supervised method varied drastically (had a high standard deviation) across bootstrapped samples, but the performance of the SSL models was more stable. The fluctuation of the purely supervised model indicated that its performance (as measured by the RMSE or other metrics) can vary significantly across different training runs or data samples. This variability can be problematic in real-world situations where there may be limited labeled data or data of varying quality. In contrast, SSL methods offer a more-stable way of learning, as the performance of the SSL models was less susceptible to fluctuations and remained more consistent across training runs or data samples. This stability is particularly useful in situations where data are limited or noisy, as it allows for more-efficient and -reliable model training. Therefore, the finding that SSL models are more stable than non-SSL models is an important contribution of this study, highlighting the potential benefits of using SSL methods in real-world applications.

## 5. Discussion

### 5.1. Implications for Mobile Sensing Systems

Mobile sensing systems have traditionally been unable to detect psychiatric and mental health outcomes such as stress due to the inherent subjectivity of the labels and variability across users. SSL on biosignals, such as the methodology introduced and evaluated here, provides an opportunity for mobile sensing solutions that detect complex psychiatric events in a personalized manner. Personalization of models is one of the leading challenges and opportunities in machine learning for healthcare and digital psychiatry [33]. From a user interaction standpoint, users are only required to provide a few (on the order of ten) labeled examples of the adverse event of interest.

Through the use of a limited number of labeled examples for the targeted adverse event, this methodology effectively reduces the user labeling burden while simultaneously facilitating the development of more-tailored and -precise models. This approach not only improves the overall user experience, but also amplifies the probability of user engagement and adherence to digital therapies due to the improved user experience required to achieve a high-performing personalized mobile sensing model.

Personalization through SSL is likely to work in other domains as well. There are numerous digital healthcare research efforts that are attempting to build machine learning models for recurring conditions and events that are traditionally infeasible for machine learning due to the subjectivity of the classes. For example, some digital therapies for pediatric autism use computer vision models to predict facial expressions evoked by conversational partners [34]. Often, such digital therapeutic systems involve the curation of user-centric data [35]. Such systems provide a clear opportunity for self-supervised personalization, as described here. Personalizing the emotion-recognition model powering such digital therapies towards frequent conversational partners by the child is possible with the methods described here.

The interaction paradigm demonstrated here is similar to how Apple’s Face ID works. Once the face recognition system is set up with only a few examples provided by the end users, it can be used repeatedly to unlock the device. Our primary goal is to demonstrate that this user interaction workflow can be adapted to mobile and wearable health settings.

In terms of implementation platforms, the HCI paradigm demonstrated here can be applied to a wide range of systems, such as smartphone apps, wearable devices, and laptops. For example, a smartphone app could use our technique to provide real-time stress monitoring and personalized tips for dealing with stress, while a wearable device could use the model to identify stress levels during physical activities or to monitor stress levels throughout the day.

The choice of platform depends on a number of factors, such as the specific use case, user preferences, and the desired amount of user activity. For example, a smartphone app might be preferred by individuals who want to actively track their stress levels and receive feedback, while a wearable device might be preferred by people who want to passively track their stress levels and have less contact.

### 5.2. Limitations and Future Work

While this work demonstrated promise for personalized learning for mobile sensing, our evaluation involved only a single dataset (WESAD) and a single modality (EDA). In future work, we would like to see our evaluation expanded to numerous biosignal datasets and health outcomes. Moving forward, an important direction for further investigation is to examine the integration of multiple signals within a single model, potentially enhancing its predictive capabilities and overall performance. Additionally, the importance of explain-ability in the medical informatics domain cannot be overstated. In future research, there is considerable potential to extend this study by exploring how different features influence our personalized model, thus enhancing its interpretability and applicability throughout the field. It is unclear whether the personalized SSL we presented will generalize to other health events that are not as strongly correlated as EDA and stress. If the method does generalize, then this work has the potential to become widely adopted in several HCI fields including wearable computing, affective computing, and mobile sensing.

There are inherent limitations to the WESAD dataset that we used for the evaluation. The WESAD dataset provides data from controlled experimental situations where realistic emotional engagement is a challenge. As the participants were required to assess their emotional state after each condition (i.e., baseline, amusement, stress, meditation), there is no continuous annotation of the participant’s emotional state in this dataset. Therefore, there is the possibility of not obtaining the participant’s actual affective state after each task. During data collection, some variables, such as a participant’s proficiency in spoken English and familiarity with the provided subject under the stress condition, may introduce bias. Also, the participants’ ages ranged from 25 to 30, which may have introduced bias into the findings.

In future work, we recommend testing the system using real-time, unimodal data obtained from participants in a more-comprehensive and -unconstrained experimental setup. This approach could potentially lead to a better understanding of the system’s performance and generalizability in real-world scenarios, further strengthening the practical applicability of our findings. Nevertheless, the WESAD dataset is a frequently used dataset in the field of affective computing using wearable data [36].

## 6. Conclusions

We presented a novel method for personalizing deep learning models for the prediction of subjective outcomes such as stress using continuous biosignals. We demonstrated the low labeling requirement for fine-tuning the model after self-supervised pre-training. This method can enable high-performance personalized modeling with minimal manual annotation effort from end users. This technique enables deep learning models to be tailored to an individual’s specific physiological reactions, resulting in more-precise and -personalized stress prediction. This has the potential to significantly increase the efficacy of stress management and preventive digital interventions while also reducing the load of the human annotation that is generally necessary for tailored models. Overall, our findings showed that self-supervised learning can enable increasingly individualized mobile sensing solutions.

## Figures and Tables

**Table 1.** Summary of literature review.

Study	Focus	Opportunities
Hannun et al. [6]	ECG deep learning	Exploration of hybrid models combining ECG with other biosignals for improved stress prediction.
Spathis et al. [11]	Speech-enabled devices	Integration of voice-based feedback mechanisms to provide real-time stress relief interventions.
Zhu et al. [12]	EDA wearables	Development of compact and user-friendly wearable devices for mass adoption.
Ninh et al. [15]	Consumer EDA devices	Design of consumer-centric applications that utilize EDA data for wellness and mental health tracking.
Eren et al. [16]	Empatica E4 device	Incorporation of multi-sensor data for a holistic health-monitoring system.
Pakarinen et al. [17]	Mental states	Broadening the scope to include the detection of other mental states like anxiety or excitement.
Kalimeri et al. [18]	Multimodal framework	Development of adaptive systems for other differently abled individuals beyond the visually impaired.
SimCLR [19] and BOYL [20]	Contrastive learning	Application of contrastive learning techniques to other images.
Poppelbaum et al. [22]	Time series analysis	Implementation in real-time systems for quick anomaly detection in biosignals.
Robert et al. [23]	EDA stress prediction	Exploration of non-invasive techniques for real-world applications.
CLOCS [24]	ECG learning	Expansion to include patient-specific health recommendations based on ECG patterns.
Sarkar et al. [26]	ECG emotion	Development of emotion-aware devices and applications for personalized user experiences.
Eldede et al. [26]	Time series learning	Application in industries like finance or meteorology where time series data are abundant.
Yang et al. [27]	TimeCLR	Incorporation of multivariate time series data for more-complex predictions.
Sunmin et al. [28]	Time series representation	Optimized personalized multimodal model
Tanvir et al. [29]	Less annotated data	Personalized multimodal machine learning model with least amount of labeled data

## Data Availability

The dataset analyzed during the current study is available at https://ubicomp.eti.uni-siegen.de/home/datasets/icmi18/, accessed on 29 October 2023.
